# A comparison between data requirements and availability for calibrating predictive ecological models for lowland UK woodlands: learning new tricks from old trees

**DOI:** 10.1002/ece3.2217

**Published:** 2016-06-17

**Authors:** Matthew R. Evans, Aristides Moustakas

**Affiliations:** ^1^ School of Biological and Chemical Sciences Queen Mary University of London Mile End Road London E1 4NS UK

**Keywords:** DBH, ecological forecasting, forestry, predictive models, SORTIE, Wytham Woods

## Abstract

Woodlands provide valuable ecosystem services, and it is important to understand their dynamics. To predict the way in which these might change, we need process‐based predictive ecological models, but these are necessarily very data intensive. We tested the ability of existing datasets to provide the parameters necessary to instantiate a well‐used forest model (SORTIE) for a well‐studied woodland (Wytham Woods). Only five of SORTIE's 16 equations describing different aspects of the life history and behavior of individual trees could be parameterized without additional data collection. One age class – seedlings – was completely missed as they are shorter than the height at which Diameter at Breast Height (DBH) is measured. The mensuration of trees has changed little in the last 400 years (focussing almost exclusively on DBH) despite major changes in the nature of the source of value obtained from trees over this time. This results in there being insufficient data to parameterize process‐based models in order to meet the societal demand for ecological prediction. We do not advocate ceasing the measurement of DBH, but we do recommend that those concerned with tree mensuration consider whether additional measures of trees could be added to their data collection protocols. We also see advantages in integrating techniques such as ground‐based LIDAR or remote sensing techniques with long‐term datasets to both preserve continuity with what has been performed in the past and to expand the range of measurements made.

## Introduction

The link between ecosystems and human society and the benefits that the latter obtain from the former are clearly acknowledged (Millennium Ecosystem Assessment 2005; TEEB 2010; DEFRA 2011). To understand the ways in which ecosystems will change, along with the goods and services that human societies obtain from them, models are needed that can be projected into the future (Clark et al. 2001; Evans 2012; Evans et al. 2012, 2013a). These models will typically be process based as these are more appropriately projected into novel conditions than statistical models. Such process‐based models are highly data intensive, and data availability may constrain the ability to develop, parameterize, and test them (Evans et al. 2014; Lonergan 2014).

Forests and woodlands are important from global to local scales and provide many goods and services – ranging from their role in the global carbon cycle to their esthetic and amenity value as well as timber. The UK's recent National Ecosystem Assessment suggested that woodlands provide many ecosystem services varying from provisioning services such as timber and fuel wood, to regulating services such as climate regulation and flood regulation, to cultural services such as recreation and tourism (DEFRA 2011). The value placed on the carbon sequestration service provided by UK woodlands was £680 million/year, with a further £77 million/year due to the carbon sequestered in harvested wood products, while the value of timber production was estimated as £113–131 million/year (DEFRA 2011; Quine et al. 2011). A more recent analysis of the economic benefit of woodlands in the UK (Economics Europe 2015) suggested that the total value of UK woodland is £270 billion.

While the language of ecosystem services has only been current for 40 years or so (Daily 1997), the goods and services provided by woodlands and forests have been valued for a long time; an example from the 18th century “forests…are of considerable service to neighbourhoods that verge upon them by furnishing them with peat and turf for their firing; with fuel for the burning of their lime; and with ashes for their grasses; and by maintaining their geese and their stock of young cattle at little or no expense” p. 24 (White 1977). As this quote illustrates, the services that were valued in the 18th century were fuel and shelter. An earlier text by John Evelyn (1665 [but presented at the Royal Society in 1662]) elaborates the many services and goods obtained from woodland in the 17th century (Evelyn 2012). These include timber for building ships, dwellings and weapons, fuel, food and shelter. Evelyn's book also details how foresters measured trees in the 17th century. Several pages (pp. 82–87) are given over to describing the sizes of particular trees in terms of the diameters and heights of their trunks and the diameter of their canopies. These measurements are then used to calculate either the amount of timber (and its value) that could be obtained from the tree or the number of animals that could be provided with shelter under its canopy. Four hundred years ago, it is clear that the main good obtained from trees was timber – it was a major construction material, and as Evelyn makes clear, the construction of ships and the security of the country depended on having good quality timber available.

The main modern reference work on forest mensuration in the UK deals with, for standing trees, the measurement of trunk diameter (DBH), basal area (area at breast height), timber height (height to which usable timber extends), tree height, and timber volume (Matthews and Mackie 2006). There is no mention of tree measurement for any purpose other than timber production in this handbook. A wide‐ranging review of this subject includes the measurements listed above and additionally includes mention of methods to measure tree crown area, crown depth, and radial growth and to estimate leaf surface area, leaf weight, and sapwood area (Laar and Akca 2007). Again there is little, if any, mention of an ecosystem service other than timber production.

DBH and tree height are the two measurements made on trees in the UK's Environmental Change Network (ECN). ECN was established in 1992 and makes standardized measurements at fixed intervals (for trees, every 3 years for DBH and every 9 years for height). ECN was one of the original members of the European network of the International Long‐Term Research network (ILTER). ILTER has adopted a comparable monitoring approach since its launch in 2003, as does the US‐LTER that has been operating since 1980. Also in 1980, the Smithsonian Tropical Research Institute (STRI) established a forest plot on Barro Colorado Island, Panama. STRI has developed tree census techniques in which all trees in a plot have DBH measured every 5 years (Condit 1998). This project has now expanded from the original plot to include over 60 plots globally (including the one referred to in this article as the Oxford plot). These comprise the Global Earth Observatory network (ForestGEO), which is now collecting data on carbon pools and fluxes on some of its plots. These various projects aimed at collecting long‐term data using forestry techniques to address ecological questions have already been extremely valuable; for a review of the ECN, see Morecroft et al. (2009) and of ForestGEO, see Anderson‐Teixeira et al. (2015).

To address concerns about the likely impact of environmental changes in the future, it will be necessary to move from describing ecosystems to predicting their likely future state in changed conditions. Ecology has not traditionally focussed on prediction despite repeated calls to do so (Simberloff 1981; Judson 1994; Grimm 1999; Clark et al. 2001; Evans et al. 2013a). For an ecosystem containing taxa with long‐lived individuals, it is almost impossible envisage how prediction can be achieved without the use of computational models.

Ecological models that are capable of being projected into the future, possibly into novel conditions outside the parameter space within which the data were collected, will have to be process based (Evans 2012; Evans et al. 2013a). The problems associated with projecting statistical models outside the bounds of the data collection are well known (Rice 2004). Process‐based models are extremely demanding of data, as there are often many interacting processes each requiring parameterization (Evans et al. 2014; Lonergan 2014). For long‐lived species, such as trees, parameterization is especially demanding as most processes occur slowly, and so require long‐term datasets to ensure that robust estimates of the relevant rates can be obtained (Moustakas et al. 2006). It is rare that datasets exist for creating such models, and so data, the collection of which was originally motivated by some other purpose, usually need to be identified and processed in a manner that makes them suitable for inclusion.

Here, we test the capability of existing data to parameterize a widely used predictive model (individual‐based model) – SORTIE (Pacala et al. 1996; Moorcroft et al. 2001; Purves and Pacala 2008; Purves et al. 2008; Strigul et al. 2008; Coomes et al. 2009; Kunstler et al. 2009; Tanentzap et al. 2013). Similar data would have been required for parameterizing other similar models, see Bugmann (2001); Snell et al. (2014) for reviews and Table 1 for summary, but we use SORTIE as an example. We have based this calibration on a well‐studied woodland – Wytham Woods in Oxfordshire, UK (Savill et al. 2010), for which we have data from a set of ECN plots and a ForestGEO plot. Our purpose was to determine whether a well‐established process‐based model could be parameterized for the UK with available data, and if not then what additional data would be required. It is therefore a test of our current capability to create models such as those that were found to be lacking by the NEA (DEFRA 2011; Evans et al. 2013a).

**Table 1:** Summary of data requirements for various four models that are alternatives to SORTIE

Model	FORMIND (Köhler and Huth 1998)	PICUS (Lexer and Hönninger 2001)	ED (Moorcroft et al. 2001)	JABOWA^a^ (Botkin 1993)
DBH	Yes	Yes	Yes	Yes
*D*_10_ of saplings	Yes		Yes	
Height	Yes	Yes	Yes	Yes
Crown height	Yes	Yes		
Crown length	Yes	Yes		
Seedlings	Yes		Yes	
Light	Yes	Yes		
Spatially explicit info (density) for neighboring dult trees	Yes	Yes	Yes	
Spatially explicit info (density) for neighboring seedlings	Yes		Yes	
In addition		Water holding capacity, initial size of soil carbon and nitrogen pools	Fecundity	Soil profile, depth of water table, soil depth, bulk density

a JABOWA contains allometric equations that have been parameterized for many North American tree species if these had to be parameterized, then much more data would be required.

## Developing a Predictive Model for Wytham Woods

### Data availability

The data that we have available are three datasets from Wytham Woods (Oxfordshire, UK) supplemented by one from Alice Holt (Hampshire, UK):

- Environmental Change Network (Wytham Woods) (ECN‐W), the ECN has monitored a fixed set of sites in Wytham Woods since 1992 (Morecroft et al. 2008)
- ECN at Alice Holt (ECN‐AH), the ECN has monitored a fixed set of sites in Alice Holt since 1994.
- Oxford University plot (OXF), a ForestGEO 18 Ha plot monitored in 2008 and 2010.
- Dawkins plots (DAW), a series of plots monitored at intervals since 1973 by Dawkins, Field, and Kirby (Dawkins and Field 1978; Horsfall and Kirby 1985; Kirby et al. 1996; Kirby 2004).

In total, there were data available on 21,614 individual trees, each of which had been measured on up to seven occasions. The data from the two ECN plots and the Oxford plot have been published elsewhere (Evans et al. 2015).

### Model's data requirements

SORTIE is an individual‐based model initially developed for the Great Mountain Forest (Connecticut) and used in USA (Pacala et al. 1996; Strigul et al. 2008), New Zealand (Coomes et al. 2009; Kunstler et al. 2009, 2011; Forsyth et al. 2015), Canada (Canham et al. 1999; Bose et al. 2015), Scotland (Tanentzap et al. 2013), and the Pyrenees (Ameztegui and Coll 2011; Ameztegui et al. 2015). It produces projections of the community structure of a forest and its carbon flux. Its basic assumptions are that trees compete for light, that adult trees grow, survive, and reproduce in relation to their size, and that saplings and seedlings grow and survive in response to their light environment. Trees are divided into three age classes – seedlings (trees <1.35 m tall), saplings (trees with DBH <10 cm), and adults (trees with DBH >10 cm). Table 2 summarizes the algorithms, parameters, and data that are required for every tree species in order to be used in SORTIE. In total, there are six pieces of data required for every seedling, five for every sapling, and seven for every adult. Examination of the data available showed:

**Table 2:** Algorithms and their parameters required to run SORTIE along with the data that are required to estimate the values of the parameters

	Submodel	Algorithm	Parameter(s)	Interpretation	Data needed for estimation
Seedling	Allometry	$H=0.1+bD_{10}$	*b* b	Slope of *H* (height) with *D*_10_ (diameter at 10 cm) relationship	Height Diameter at 10 cm
	Growth	$G_{\mathrm{seed}}=\left(\alpha L/\left(L+\left(\alpha /\beta \right)\right)\right)D_{10}^{\phi }$	*α* b	Asymptotic *G*_seed_ (seedling diameter growth rate) light relationship	Diameter at 10 cm on at least two occasions to estimate *G*_seed_
			*β* b	Slope of *G*_seed_ light relationship in low light	Proportion of ambient light reaching tree
			*ϕ* b	Exponent of the *G*_seed_ – *D*_10_ relationship	
	Mortality	$p\left(H,L\right)=M_{\max}e^{\left(-aH^{b}-cL^{d}\right)}$	*a* c		Height
			*b* c	Modifier of height effect	Proportion of ambient light reaching tree
			*c* c		Maximum recorded annual mortality rate (*M*_max_)
			*d* c	Modifier of light effect	Mortality (*M*)
	Dispersal	$R_{i}=\text{STR}\sum_{j=1}^{n}\left({\left(\frac{{\text{DBH}}_{j}}{30}\right)}^{\beta }e^{-Dd_{ji}^{\theta }}\right)$ or $R_{i}=\text{STR}\sum_{j=1}^{n}\left({\left(\frac{{\text{DBH}}_{j}}{30}\right)}^{\beta }e^{-0.5{\left(\frac{\ln(d_{ij}/x_{0})}{x_{b}}\right)}^{2}}\right)$	STR^d^	Standardized total recruits (number of seedlings produced by a 30‐cm DBH tree)	Density of seedlings at a point *i* (*R*_*i*_)
			*D* c	Species‐specific dispersal parameter	Diameter at Breast Height of parent trees
			*θ* c	Dispersal parameter	
			*β* c	Dispersal parameter	
			*x*_0_ c	Mean of the log normal function	
			*x*_b_ c	Variance of the log normal function	
Sapling	Allometry	$\text{DBH}=a+bD_{10}$	*a* b	DBH when *D*_10_ is zero	Diameter at Breast Height
			*b* b	Slope of DBH with *D*_10_ relationship	Diameter at 10 cm
		$H=aD_{10}^{b}$	*a* b	Slope of H with *D*_10_ relationship	Height
			*b* b	Exponent of relationship between *H* and *D*_10_	Diameter at 10 cm
	Growth	$G_{sap}=\left(\alpha L/\left(L+\left(\alpha /\beta \right)\right)\right)D_{10}^{\phi }$	*α* b	Asymptotic *G*_sap_ (sapling diameter growth rate) light relationship	Diameter at 10 cm on at least two occasions to estimate *G*_sap_
			*β* b	Slope of *G*_sap_ light relationship in low light	Proportion of ambient light reaching tree
			*ϕ* b	Exponent of the *G*_sap_ – *D*_10_ relationship	
	Mortality^a^	$\begin{array}{cc} & P_{\mathrm{mort}}=1-\left\{\left\{e^{\left(\beta 1+\left(\left(\beta 2\left(\text{DBH}-\bar{\text{DBH}}\right)\right)s_{\text{DBH}}\right)\right)}\right\}\right.\\ & \left.÷\left\{1+e^{\left(\beta 1+\left(\left(\beta 2\left(\text{DBH}-\bar{\text{DBH}}\right)\right)s_{\text{DBH}}\right)\right)}\right\}\right\} \end{array}$	*β*1^b^	Intercept of the logit function relating probability of survival to DBH	Diameter at Breast Height
			*β*2^b^	Slope of the logit function relating probability of survival to DBH	Survival
Adult	Allometry	$\text{CRad}=a{\text{DBH}}^{b}$	*a* b	Slope of CRad (crown radius) – DBH relationship	Crown radius (CRad)
			*b* b	Exponent of the relationship between CRad and DBH	Diameter at Breast Height
		$\text{CH}=aH^{b}$	*a* b	Slope of CH (crown height) – *H* relationship	Crown Height (CH)
			*b* b	Exponent of the relationship between CH and *H*	Height
		$H=1.35+\left(\max H-1.35\right)\left(1-e^{-b\text{DBH}}\right)$	*b* b	Slope of *H* with DBH relationship	Height
					Diameter at Breast Height
					Maximum height (max*H*)
	Growth	$G_{\text{adult}}=\text{Max}G\times \text{SE}\times \text{CE}$			Diameter at Breast Height
		$\text{SE}=e^{-0.5{\left(\ln\left(\text{DBH}/x_{0}\right)/x_{b}\right)}^{2}}$	SE, which requires: *x*_0_ c *x*_b_ c	Devaluation of Max*G* (maximum adult growth rate) by size	DBH on at least two occasions to estimate annual diameter growth rate (*G*_adult_)
		$\text{CE}=e^{-C{\left(\text{BA}{}_{\mathrm{supp}}/1000\right)}^{D}}$	CE, which requires: *C* c *D* c	Devaluation of Max*G* by crowding	Maximum annual diameter growth rate (Max*G*)
					Basal area of trees larger than target tree within 400 m^2^ (BA_supp_)
	Light				Canopy openness
	Mortality^a^	$\begin{array}{cc} & P_{\mathrm{mort}}=1-\left\{\left\{e^{\left(\beta 1+\left(\left(\beta 2\left(\text{DBH}-\bar{\text{DBH}}\right)\right)s_{\text{DBH}}\right)\right)}\right\}\right.\\ & \left.÷\left\{1+e^{\left(\beta 1+\left(\left(\beta 2\left(\text{DBH}-\bar{\text{DBH}}\right)\right)s_{\text{DBH}}\right)\right)}\right\}\right\} \end{array}$	*β*1^b^	Intercept of the logit function relating probability of survival to DBH	Diameter at Breast Height
			*β*1^b^	Slope of the logit function relating probability of survival to DBH	Survival

a We have departed from the usual SORTIE functions for mortality as a result of our empirical investigations that demonstrated that tree survival was better predicted by size than by light or growth rate (Moustakas and Evans 2015).

b Derived by statistical analysis of data.

c Derived by inverse modeling.

d Derived by comparison of data with model output.

1. Seedlings: No data exist in any of the available datasets on trees that are shorter than the height at which DBH is measured. Therefore, no seedlings could be included in SORTIE UK.
2. Saplings: DBH data exist in all datasets and height in the ECN datasets. Therefore, it was possible to estimate growth rate but this could not be related to light environment without light data. An alternative would be to use SORTIE's capability to estimate the light environment at any specific location. To achieve this, the trees would need to be mapped accurately and canopy openness would need to be known. Using the DBH records as records of presence, we have been able to estimate mortality (Moustakas and Evans 2015). However, as the allometric relationships of saplings all relate to diameter at 10 cm above ground (*D*_10_) and this is not included in the available datasets, no allometric relationships could be derived. This meant the height data were not useable without further data collection.
3. Adults: DBH exists in all datasets, and height data exist in the ECN datasets. Therefore, it was possible to fully parameterize the height – DBH allometric equation but the two other allometric equations could not be parameterized. We could use the DBH records to estimate growth rate, and we were able to estimate the effect of size on growth rate. As with saplings, we have been able to estimate mortality using DBH records as records of presence (Moustakas and Evans 2015).

Therefore, using the available data, we were able to parameterize five of the 16 equations required to run SORTIE. Over the last 4 years, we have collected the missing data from all the ECN trees and a subset of the trees in OXF (Evans et al. 2015), and we are now in a position to use SORTIE at Wytham Woods.

### Data volumes

The data demands outlined in the previous section need to be met for each tree species that one wishes to include in the model. We have focussed our efforts on the eight commonest deciduous species in Wytham that between them represent over 99% of all individuals. Even for these common species, the total number of usable data records for each species remains low (Table 3). The reasons for this are the following: Firstly, to estimate the parameters for any relationship usually at least two different pieces of data are needed for each individual (either data on two different variables, e.g., DBH and height or measurements of the same variable at two different time points), thus reducing the number of usable data below the sample size that might exist for either individually. Secondly, each species has to be parameterized for all age classes separately, which obviously reduces the sample size available for any age class below the total for the species. This means that at present, of the 21,614 trees in the datasets, only 726 have provided complete data for the production of the model.

**Table 3:** The numbers of individuals of each species from each dataset that have been included in any analysis to estimate the parameters for SORTIE

	Adults				Saplings			
	ECN‐AH	ECN‐W	OXF	Total	ECN‐AH	ECN‐W	OXF	Total
Field maple	4	13		17	0	2	4	6
Sycamore	0	50		50	0	17	21	38
Birch	23	21		44	39	1	0	40
Hazel	10	22		32	34	17	14	65
Hawthorn	6	19		25	13	8	16	37
Beech	3	23		26	0	3	16	19
Ash	22	51		73	15	18	15	48
Oak	132	21		153	44	7	2	53
Total	200	220	0	420	145	73	88	306

## Discussion

It is perhaps unsurprising that the data collected by ECN and ForestGEO at their plots in Wytham Woods do not allow the parameterization of the model we have used. These surveys were not set up with this purpose and so should not be criticized for not meeting its needs. However, while it may seem elementary that “plants stand still to be counted and do not have to be trapped, shot, chased, or estimated” (Harper 1977), monitoring and analyzing long‐term datasets often involves accounting for data collected for other purposes. Today we measure trees in much the same way as that recorded by Evelyn in the middle of the 17th century (Evelyn 2012), and the main measurement is a convenient diameter of the tree at some distance up its trunk (Matthews and Mackie 2006). The use of this measurement seems to be ubiquitous among those involved with monitoring woodlands (Hiley 1954; Matthews and Mackie 2006), but it is important to ask why we are using it, and whether it continues to be of utility. Given the huge changes in the relative values of the goods and services provided by woodlands to society over 350 years, it is perhaps surprising that our methods of tree mensuration have changed so little.

It is the case that Wytham Woods are well studied and has good data availability; it is likely that if models cannot be parameterized at this location, they will be difficult to parameterize for any site in the UK without additional data collection. One criticism of what we have carried out could be that we are attempting to use a model that is unusually demanding of data. However, competing models are broadly comparable with SORTIE (Table 1, Bugmann (2001); Snell et al. (2014). The rationale of our work is that there is a societal demand for projections of the future state of ecosystems (DEFRA 2011). To meet this demand, several key issues need to be addressed – principally the modeling framework and data availability. At present, the data even at a relatively well‐studied location do not seem to be adequate to parameterize models that could be used to project the state of ecosystems into the future. To achieve this, there would seem to be a need to bring modeling and measurement closer together and to allow the former without jeopardizing the latter. It is hoped that this article can be seen as an attempt to do so.

**Figure 1:**
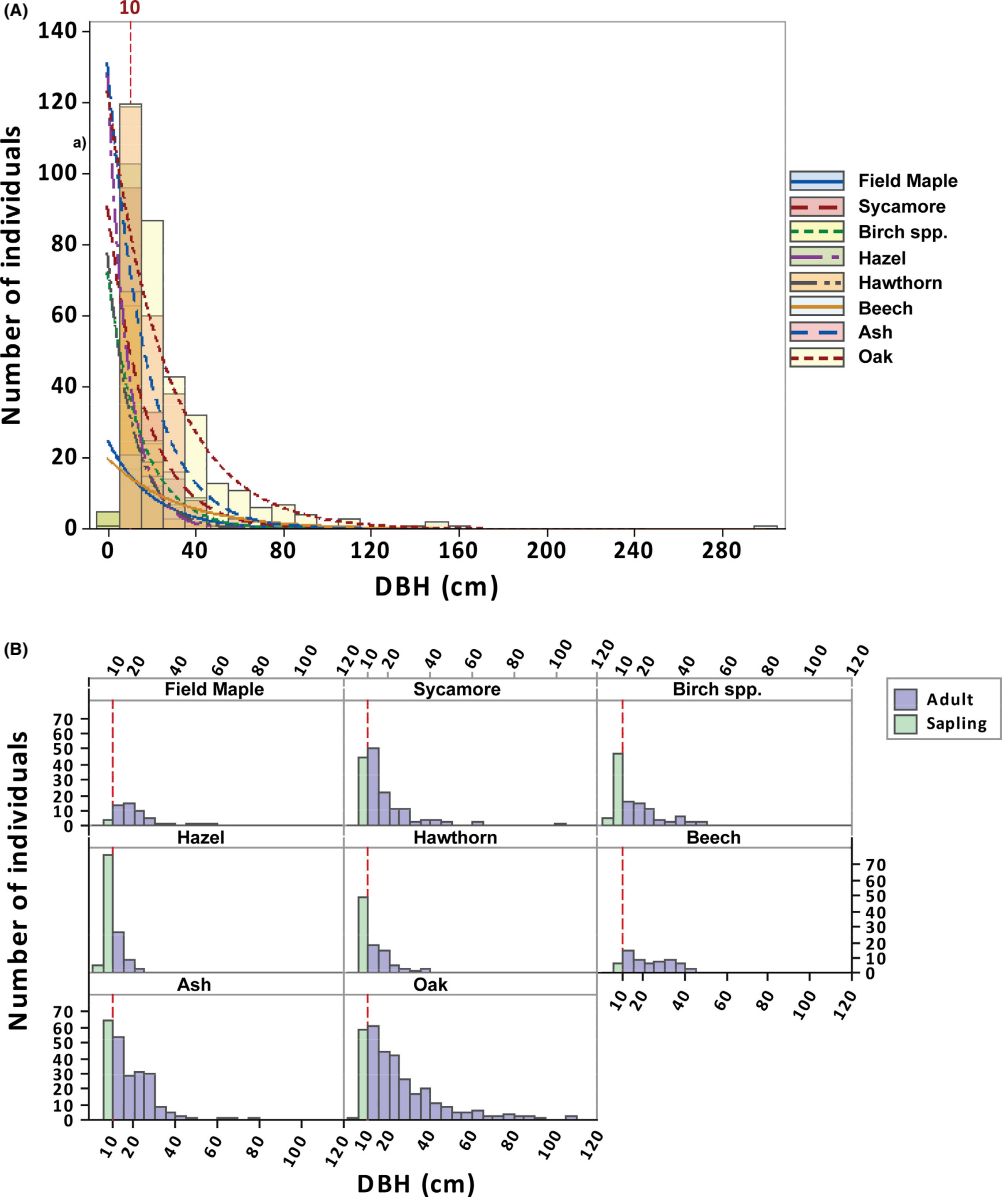
(A) Size class distribution in terms of DBH in cm of eight tree species in Wytham Wood, with a negative exponential distribution overlaid for each species. The vertical line at 10 cm corresponds to the maximum size of saplings. (B) Species‐specific detail of size class distributions in terms of DBH of the eight tree species. For most species, there are fewer saplings than you would expect and no seedlings, the vertical red line marks the upper size limit for saplings. This makes it hard to assess whether there is lack of recruitment and impossible to calibrate predictive models.

The use of any measurement of tree size that is taken at some distance from the ground will automatically exclude any individual that is shorter than the height at which this measurement is made, and this is why there are no data on seedlings in any dataset to which we have access. Obviously, the higher the measurement is taken, the greater the number of individuals that will be excluded. This is shown in Figure 1, which demonstrates that a substantial number of individuals are predicted to exist below the currently recorded minimum size classes. Seedling data from ECN taken within the same plots on which larger trees are measured reveal that for some species, seedlings make up a very large fraction of the individuals in the population – for example, for ash, 26% of all individuals are seedlings (Table 4). Overall, the lack of lower size classes is a serious data omission not only for predictive modeling calibration but also for forest assessment as it is impossible to detect whether there is a lack of recruitment for some species.

**Table 4:** Number of seedlings, sapling, and adults trees per hectare of each species in Wytham Woods, seedling information calculated from ECN data recorded in 0.4 × 0.4 m quadrats

Species	Seedlings/Ha	Saplings/Ha	Adults/Ha
Field maple	457	2440	1733
Sycamore	7431	158,672	170,035
Birch	305	257	2407
Hazel	38	49,274	6805
Hawthorn	2896	37,557	6035
Beech	114	2761	3724
Ash	58,727	72,354	98,131
Oak	152	128	11,685

The second point worth noting about DBH is that for modeling purposes, it could be replaced with any trunk diameter measurement. All relationships that use DBH could use any available trunk diameter including *D*_10_. Had there been good data on *D*_10_ for all species, then all allometric equations could be related to *D*_10_ rather than DBH, which would then be redundant. There are many reasons to continue measuring DBH, it is simple, ergonomically undemanding, and can be assessed with greater accuracy than measurements lower on trunks. These reasons in addition to the long‐term datasets that have been created justify continuing the measurement of DBH, but we would argue for new measurements to be added.

**Figure 2:**
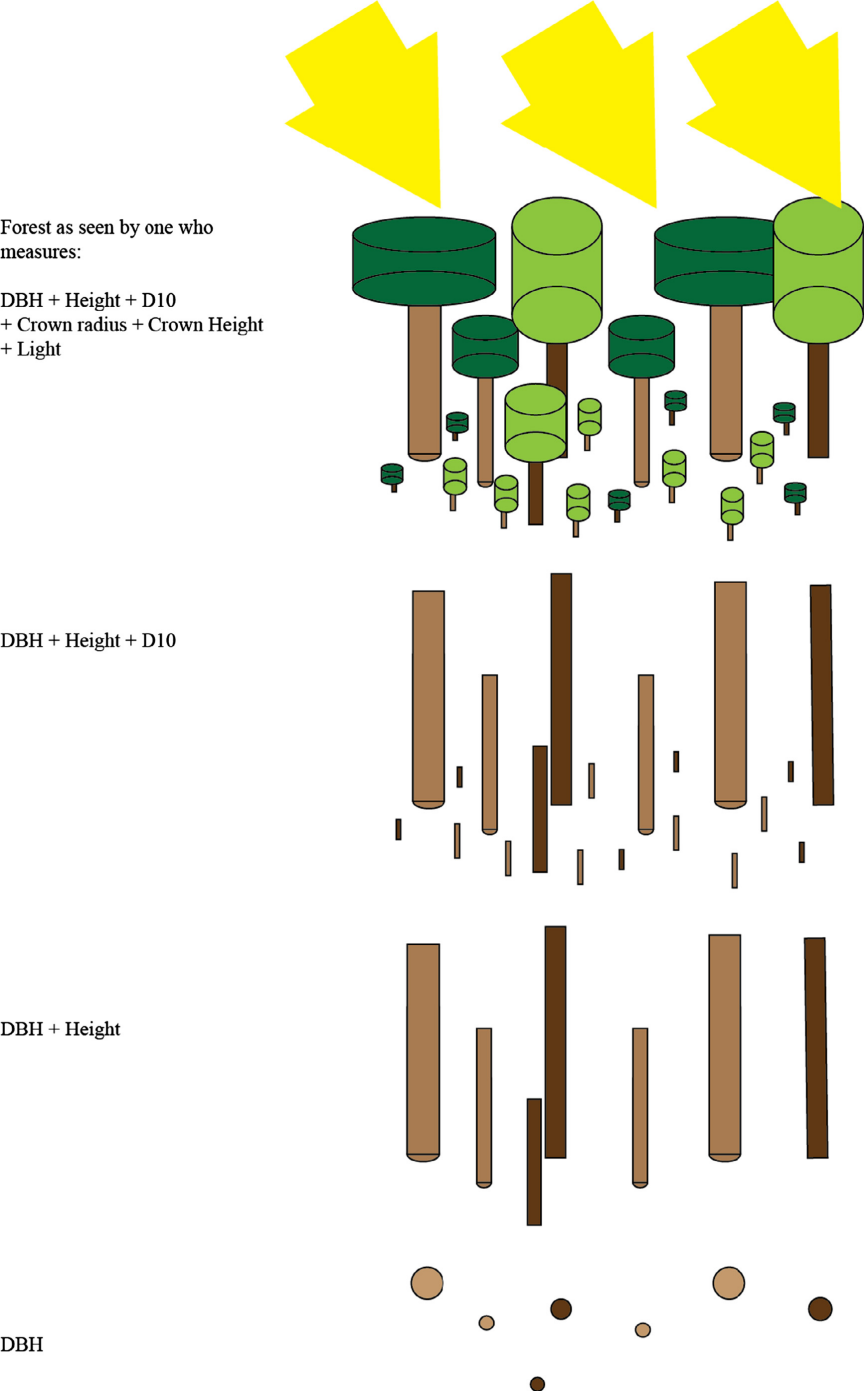
The nature of the modeled forest and hence our ability to both predict and understand it increases in realism and complexity as data on more parameters are concerned. If we consider DBH alone (bottom), then the forest is simply a series of trunks in cross section, if additionally include height, *D*_10_, crown measurements, and light, then a simplified but recognizable forest appears.

The final point emerging from this analysis is that measuring only DBH with no other measurements allows very few parameters to be estimated. We have shown that it is possible to use DBH records to assess the mortality of trees (Moustakas and Evans 2015), but other than that, little parameter estimation is possible – only annual diameter growth rate and the effect of size on growth rate can be estimated, and then, only in adults if light data are missing for saplings. However, note that the light data can be estimated from SORTIE given information on canopy openness and tree positions. ECN has measured tree height in addition to DBH, and this allows the parameters of the height‐DBH equation to be estimated for adults. The variables that most increase the ability to calibrate allometric equations and thus develop predictive models are *D*_10_ and light (Table 5). These both add an additional three parameterizable equations to the ones that can be parameterized with DBH and height. Figure 2 illustrates how the realism of the modeled forest increases as additional variables are considered. It is inevitable that as one includes additional information, then one can achieve greater realism, but active decisions should be made about how to trade off realism against the costs of data collection, processing, and simulation (Evans et al. 2013b; Weisberg 2013).

**Table 5:** The number of equations that can be successfully parameterized increases with the number of different types of data available

Data available for:	Cumulative number of equations for which parameters can be estimated		
	Seedlings	Saplings	Adults
DBH	0	1	3
DBH + Height	0	1	4
DBH + Height + *D*_10_	1	3	4
DBH + Height + *D*_10_ + Light	3	4	4
DBH + Height + *D*_10_ + Light + Crown height	3	4	5
DBH + Height + *D*_10_ + Light + Crown height + Crown radius	3	4	6
DBH + Height + *D*_10_+ Crown height + Crown radius + Light +Canopy openness	3	4	7
DBH + Height + *D*_10_ + Crown height + Crown radius + Light + Canopy openness + Basal area of surrounding trees	3	4	8
DBH + Height + *D*_10_ + Crown height + Crown radius + Light + Canopy openness + Basal area of surrounding trees + Seedling density	4	4	8

We are not advocating an end to measuring DBH. If we were to do so the valuable long‐term datasets that have been built up to now would be lost. At the very least, an alternative measurement protocol would need to run alongside one using DBH for a period of time to allow the interconversion of one set of measurements to the other. What we do suggest is giving some thought to the addition of other measurements to standard protocols. In the relatively near future, it is likely that the utility of remote sensing data will increase to a point where it would be desirable to integrate remotely sensed data with these long‐term datasets. An example would be the ability of a ground‐based LIDAR system to measure DBH, basal area, woody biomass, stand height, foliage profile, crown diameter, and stem count (Yao et al. 2011; Zhao et al. 2011; Yang et al. 2013). This would seem to be a cost‐effective means to collect detailed information on woodland structure, but repeated surveys would be needed to build up adequate data to assess all parameters and the way in which they change with time. The recently announced European Space Agency Biomass satellite may provide a further option. Biomass will use P‐band synthetic aperture radar (420–450 MHz) to map the woody parts of forests with a return rate of 6 months over a projected 5‐year period. Unfortunately, due to conflicts with military radar use, this will apparently not be available northern parts of North America or Europe, including the UK.

If it matters to society that we understand how ecosystems and the services they provide might change into the future, then we need the data to develop the models to do so (Evans et al. 2014; Lonergan 2014). To project systems into the future, into potentially unknown conditions, process‐based models are needed (Evans et al. 2012, 2013a). By their nature these are demanding of data, datasets (even ones of long duration) will be of no utility if they only contain data on a single variable. Our conclusion is that despite the huge efforts that have gone into measuring and recording data, the data that were available in this ecosystem at the start of this project were insufficient to parameterize a widely used process‐based model. To do so, a significant amount of additional effort was required even for this well‐studied ecosystem. This means that it will be difficult, without substantial additional data collection, to develop the models required to make projections of woodland ecosystems that were felt to be desirable by, for example, the UK's National Ecosystem Assessment (DEFRA 2011).

## Conflict of Interest

None declared.

## References

[ece32217-bib-0001] Ameztegui, A. , and L. Coll . 2011 Tree dynamics and co‐existence in the montane‐sub‐alpine ecotone: the role of different light‐induced strategies. J. Veg. Sci. 22:1049–1061.

[ece32217-bib-0002] Ameztegui, A. , L. Coll , and C. Messier . 2015 Modelling the effect of climate‐induced changes in recruitment and juvenile growth on mixed‐forest dynamics: the case of montane‐subalpine Pyrenean ecotones. Ecol. Model. 313:84–93.

[ece32217-bib-0003] Anderson‐Teixeira, K. J. , S. J. Davies , A. C. Bennett , E. B. Gonzalez‐Akre , H. C. Muller‐Landau , S. Joseph Wright , et al. 2015 CTFS‐ForestGEO: a worldwide network monitoring forests in an era of global change. Glob. Change Biol. 21:528–549. 10.1111/gcb.12712 25258024

[ece32217-bib-0004] Bose, A. K. , B. D. Harvey , K. D. Coates , S. Brais , and Y. Bergeron . 2015 Modelling stand development after partial harvesting in boreal mixedwoods of eastern Canada. Ecol. Model. 300:123–136.

[ece32217-bib-0005] Botkin, D. B. 1993 Forest dynamics: an ecological model. Oxford University Press, New York, NY.

[ece32217-bib-0006] Bugmann, H. 2001 A review of forest gap models. Clim. Change. 51:259–305.

[ece32217-bib-0007] Canham, C. D. , K. D. Coates , P. Bartemucci , and S. Quaglia . 1999 Measurement and modeling of spatially‐explicit variation in light transmission through interior cedar‐hemlock forests of British Columbia. Can. J. For. Res. 29:1775–1783.

[ece32217-bib-0008] Clark, J. S. , S. R. Carpenter , M. Barber , S. Collins , A. Dobson , J. A. Foley , et al. 2001 Ecological forecasts: an emerging imperative. Science 293:657–660. 11474103 10.1126/science.293.5530.657

[ece32217-bib-0009] Condit, R. 1998 Tropical forest census plots: methods and results from Barro Colorado Island, Panama and a comparison with other plots. Springer, Berlin, Germany.

[ece32217-bib-0010] Coomes, D. A. , G. Kunstler , C. D. Canham , and E. Wright . 2009 A greater range of shade‐tolerance niches in nutrient‐rich forests: an explanation for positive richness–productivity relationships? J. Ecol. 97:705–717.

[ece32217-bib-0011] Daily, G. C. 1997 Nature's services; societal dependence on natural ecosystems. Island Press, Washington, DC.

[ece32217-bib-0012] Dawkins, H. C. D. , and D. R. B. Field . 1978 A long‐term surveillance system for British woodland vegetation. Commonwealth Forestry Institute, Oxford.

[ece32217-bib-0013] DEFRA . 2011 The UK National Ecosystem Assessment: synthesis of the key findings. UNEP‐WCMC, Cambridge, UK.

[ece32217-bib-0014] Economics Europe . 2015 P. 28 *in* The economic benefits of woodland. Woodland Trust, Grantham, UK.

[ece32217-bib-0015] Evans, M. R. 2012 Modelling ecological systems in a changing world. Philos. Trans. R. Soc. Lond. B Biol. Sci. 367:181–190. 22144381 10.1098/rstb.2011.0172 PMC3223798

[ece32217-bib-0016] Evans, M. R. , K. Norris , and T. G. Benton . 2012 Predictive ecology: systems approaches. Philos. Trans. R. Soc. Lond. B Biol. Sci. 367:163–169. 22144379 10.1098/rstb.2011.0191 PMC3223810

[ece32217-bib-0017] Evans, M. R. , M. Bithell , S. J. Cornell , S. R. X. Dall , S. Diaz , S. Emmott , et al. 2013a Predictive systems ecology. Proc. R. Soc. B Biol. Sci. 280:20131452. 10.1098/rspb.2013.1452 PMC3790477 24089332

[ece32217-bib-0018] Evans, M. R. , V. Grimm , K. Johst , T. Knuuttila , R. De Langhe , C. M. Lessells , et al. 2013b Do simple models lead to generality in ecology? Trends Ecol. Evol. 28:578–583. 23827437 10.1016/j.tree.2013.05.022

[ece32217-bib-0019] Evans, M. R. , T. G. Benton , V. Grimm , C. M. Lessells , M. A. O'Malley , A. Moustakas , et al. 2014 Data availability and model complexity, generality, and utility: a reply to Lonergan. Trends Ecol. Evol. 29:302–303. 24709222 10.1016/j.tree.2014.03.004

[ece32217-bib-0020] Evans, M. R. , A. Moustakas , G. Carey , Y. Malhi , N. Butt , S. Benham , et al. 2015 Allometry and growth of eight tree taxa in United Kingdom woodlands. Sci. Data 2:150006. 25977813 10.1038/sdata.2015.6 PMC4413227

[ece32217-bib-0021] Evelyn, J. 2012 Sylva; or a discourse of forest‐trees, and the propagation of timber in his majesties dominions. Forgotten Books, London, UK.

[ece32217-bib-0022] Forsyth, D. M. , D. J. Wilson , T. A. Easdale , G. Kunstler , C. D. Canham , W. A. Ruscoe , et al. 2015 Century‐scale effects of invasive deer and rodents on the dynamics of forests growing on soil of contrasting fertility. Ecol. Monogr. 85:157–180.

[ece32217-bib-0023] Grimm, V. 1999 Ten years of individual‐based modelling in ecology: what have we learned and what could we learn in the future? Ecol. Model. 115:129–148.

[ece32217-bib-0024] Harper, J. L. 1977 Population biology of plants. Academic Press, London.

[ece32217-bib-0025] Hiley, W. E. 1954 Woodland management, 1st ed Faber and Faber, London, UK.

[ece32217-bib-0026] Horsfall, A. S. , and K. J. Kirby . 1985 The use of permanent plots to record changes in the structure and composition of Wytham Woods. Research and Survey in Nature Conservation 1 Nature Conservancy Council, Peterborough.

[ece32217-bib-0027] Judson, O. P. 1994 The rise of the individual‐based model in ecology. Trends Ecol. Evol. 9:9–14. 21236754 10.1016/0169-5347(94)90225-9

[ece32217-bib-0028] Kirby, K. J. 2004 Changes in ground flora in Wytham Woods, southern England, 1974‐2002, in stands of different origins and past treatment Pp. 193–203 *in* Honnay O., Verheyen K., Bossuyt B. and Hermy M., eds. Forest biodiversity: lessons from history for conservation. CABI, Wallingford.

[ece32217-bib-0029] Kirby, K. J. , R. C. Thomas , and H. C. D. Dawkins . 1996 Monitoring of changes in tree and shrub layers in Wytham Woods (Oxfordshire), 1974‐1991. Forestry 69:319–334.

[ece32217-bib-0030] Köhler, P. , and A. Huth . 1998 The effects of tree species grouping in tropical rain forest modelling. Ecol. Model. 109:301–321.

[ece32217-bib-0031] Kunstler, G. , D. A. Coomes , and C. D. Canham . 2009 Size‐dependence of growth and mortality influence the shade tolerance of trees in a lowland temperate rain forest. J. Ecol. 97:685–695.

[ece32217-bib-0032] Kunstler, G. , R. B. Allen , D. A. Coomes , C. D. Canham , and E. F. Wright . 2011 SORTIE/NZ model development. Landcare Research New Zealand Ltd, Lincoln.

[ece32217-bib-0033] Laar, A. V. , and A. Akca . 2007 Forest mensuration, 2nd ed Springer, Dordrecht, Netherlands.

[ece32217-bib-0034] Lexer, M. J. , and K. Hönninger . 2001 A modified 3D‐patch model for spatially explicit simulation of vegetation composition in heterogeneous landscapes. For. Ecol. Manage. 144:43–65.

[ece32217-bib-0035] Lonergan, M. 2014 Data availability constrains model complexity, generality, and utility: a response to Evans et al. Trends Ecol. Evol. 29:301–302. 24703902 10.1016/j.tree.2014.03.005

[ece32217-bib-0036] Matthews, R. W. , and E. D. Mackie . 2006 Forest mensuration: a handbook for practitioners. Forestry Commission, Edinburgh, UK.

[ece32217-bib-0037] Millennium Ecosystem Assessment . 2005 Ecosystems and human well‐being: synthesis. Island Press, Washington, DC.

[ece32217-bib-0038] Moorcroft, P. R. , G. C. Hurtt , and S. W. Pacala . 2001 A method for scaling vegetation dynamics: the ecosystem demography model (ED). Ecol. Monogr. 71:557–586.

[ece32217-bib-0039] Morecroft, M. , V. J. Stokes , M. E. Taylor , and J. I. L. Morison . 2008 Effects of climate and management history on the distribution and growth of sycamore (*Acer pseudoplantanus* L.) in a osuthern British woodland in comparison to native competitors. Forestry 81:59–74.

[ece32217-bib-0040] Morecroft, M. D. , C. E. Bealey , D. A. Beaumont , S. Benham , D. R. Brooks , T. P. Burt , et al. 2009 The UK Environmental Change Network: emerging trends in the composition of plant and animal communities and the physical environment. Biol. Conserv. 142:2814–2832.

[ece32217-bib-0041] Moustakas, A. , and M. R. Evans . 2015 Effects of growth rate, size, and light availability on tree survival across life stages: a demographic analysis accounting for missing values and small sample sizes. BMC Ecol. 15:6, doi: 10.1186/s12898‐015‐0038‐8. 10.1186/s12898-015-0038-8 PMC4465470 25886407

[ece32217-bib-0042] Moustakas, A. , M. Guenther , K. Wiegand , K.‐H. Mueller , D. Ward , K. M. Meyer , et al. 2006 Long‐term mortality patterns of the deep‐rooted *Acacia erioloba*: the middle class shall die!. J. Veg. Sci. 17:473–480.

[ece32217-bib-0043] Pacala, S. W. , C. D. Canham , J. Saponara , J. A. Silander , R. K. Kobe , and E. Ribbens . 1996 Forest models defined by field measurements: estimation, error analysis and dynamics. Ecol. Monogr. 66:1–43.

[ece32217-bib-0044] Purves, D. , and S. Pacala . 2008 Predictive models of forest dynamics. Science 320:1452–1453. 18556548 10.1126/science.1155359

[ece32217-bib-0045] Purves, D. W. , J. W. Lichstein , N. Strigul , and S. W. Pacala . 2008 Predicting and understanding forest dynamics using a simple tractable model. Proc. Natl Acad. Sci. USA 105:17018–17022. 18971335 10.1073/pnas.0807754105 PMC2579370

[ece32217-bib-0046] Quine, C. , C. Cahalan , A. Hester , J. Humphrey , J. Kirby , A. Moffat , et al. 2011 Woodlands. The UK National Ecosystem Assessment Technical Report. UK National Ecosystem Assessment UNEP‐WCMC, Cambridge, UK.

[ece32217-bib-0047] Rice, K. 2004 Sprint research runs into a credibility gap. Nature 432:147. 10.1038/432147b 15538340

[ece32217-bib-0048] Savill, P. S. , C. M. Perrins , K. J. Kirby , and N. Fisher . 2010 Wytham Woods: Oxford's ecological laboratory. Oxford University Press, Oxford, UK.

[ece32217-bib-0049] Simberloff, D. 1981 The sick science of ecology. Eidema 1:49–54.

[ece32217-bib-0050] Snell, R. S. , A. Huth , J. E. M. S. Nabel , G. Bocedi , J. M. J. Travis , D. Gravel , et al. 2014 Using dynamic vegetation models to simulate plant range shifts. Ecography 37:1184–1197.

[ece32217-bib-0051] Strigul, N. , D. Pristinski , D. Purves , J. Dushoff , and S. Pacala . 2008 Scaling from trees to forests: tractable macroscopic equations for forest dynamics. Ecol. Monogr. 78:523–545.

[ece32217-bib-0052] Tanentzap, A. J. , J. Zou , and D. A. Coomes . 2013 Getting the biggest birch for the bang: restoring and expanding upland birchwoods in the Scottish Highlands by managing red deer. Ecol. Evol. 3:1890–1901. 23919137 10.1002/ece3.548 PMC3728932

[ece32217-bib-0053] TEEB . 2010 The economics of ecosystems and biodiversity ecological and economic foundations. Earthscan, London.

[ece32217-bib-0054] Weisberg, M. 2013 Simulation and similarity: using models to understand the world. Oxford University Press, Oxford, UK.

[ece32217-bib-0055] White, G. 1977 The natural history and antiquities of Selborne in the county of Southampton. Penguin Classics, London, UK.

[ece32217-bib-0056] Yang, X. , A. H. Strahler , C. B. Schaaf , D. L. B. Jupp , T. Yao , F. Zhao , et al. 2013 Three‐dimensional forest reconstruction and structural parameter retrievals using a terrestrial full‐waveform LIDAR instrument (Echidna^®^). Remote Sens. Environ. 135:36–51.

[ece32217-bib-0057] Yao, T. , X. Yang , F. Zhao , Z. Wang , Q. Zhang , D. Jupp , et al. 2011 Measuring forest structure and biomass in New England forest stands using Echidna ground‐based LIDAR. Remote Sens. Environ. 115:2965–2974.

[ece32217-bib-0058] Zhao, F. , X. Yang , M. A. Schull , M. O. Román‐Colón , T. Yao , Z. Wang , et al. 2011 Measuring effective leaf area index, foliage profile, and stand height in New England forest stands using a full‐waveform ground‐based LIDAR. Remote Sens. Environ. 115:2954–2964.

